# Anti-hepatocellular carcinoma activity of *Jacaranda mimosifolia* through experimental validation and network pharmacology

**DOI:** 10.1371/journal.pone.0346325

**Published:** 2026-04-03

**Authors:** Ayesha Bibi, Muhammad Hamza Afandi, Azra Mehmood, Usman Ali Ashfaq, Muhammad Shareef Masoud, Mohsin Ahmad Khan, Rashid Bhatti

**Affiliations:** 1 Centre of Excellence in Molecular Biology, University of the Punjab, Lahore, Pakistan; 2 Department of Bioinformatics and Biotechnology, Faculty of Life Sciences, Government College University, Faisalabad, Pakistan; Rutgers: Rutgers The State University of New Jersey, UNITED STATES OF AMERICA

## Abstract

Hepatocellular carcinoma (HCC) has a very significant mortality rate and is one of the most common cancers worldwide. *Jacaranda mimosifolia* is reported to have potential antitumor activities against various human cancers. However, the effects of *J. mimosifolia* on HCC are yet elusive. This study aimed to investigate the anti-HCC potential of methanolic extract of *J. mimosifolia* leaves using *in vitro* and *in vivo* studies and a network pharmacology approach. The effect of *J. mimosifolia* extract was assessed on Huh-7.5 cells using MTT assay, wound healing assay, and DNA fragmentation assay. These experiments found that *J. mimosifolia* extract significantly suppressed Huh-7.5 cell proliferation, impaired cell migration, and induced cell apoptosis. The real-time PCR validated the upregulation of *p53* and *Bax*, alongside the downregulation of *AFP* and *GPC3 in* Huh-7.5 cells after treatment with *J. mimosifolia* extract. *In vivo* experiments confirmed the hepatoprotective effects of *J. mimosifolia* extract in mice models with CCl_4_-induced hepatic injury. In addition, through network pharmacological analysis, *J. mimosifolia* was found to play a critical role against HCC via targeting multiple potential targets and pathways. Docking analysis identified apigenin and kaempferol with the lowest binding energy against PTGS2 and EGFR, respectively, while flavonol glycoside showed the lowest binding energy against MMP9. However, detailed research is needed to isolate the potential phytochemicals from *J. mimosifolia* against HCC.

## Introduction

Liver cancer is the third most common type of cancer, followed by lung and stomach cancer [1]. Among 9,555,027 cancer-related deaths, 781,631 cases (8.18%) died of liver cancer [2]. Hepatitis B, hepatitis C, metabolic syndrome, obesity, diabetes, alcohol, nonalcoholic fatty liver disease, Aflatoxin B_1,_ tobacco, and familial genetic susceptibility have increased the risk of liver cancer [3]. Hepatocellular carcinoma (HCC) is the most common subtype of primary liver cancer that accounts for 75% of all liver cancers [4]. It is the sixth most common cancer throughout the world, ranking fifth in men and eighth in women [5]. In 2012, more than 782,000 cases of HCC were estimated to occur, as reported by the IARC (International Agency for Research on Cancer) [6]. Hormone therapy, radiotherapy, chemotherapy, and surgery are considered possible treatments for HCC. However, all these therapies have toxic effects on an individual’s healthy cells and destroy them non-specifically [7].

Plant secondary metabolites possess diverse biological properties such as anti-inflammatory, anti-cancer, and anti-microbial [8]. Alkaloids, flavonoids, phenols, and terpenoids are some secondary metabolites observed for their anti-cancer activities and may therefore be further developed as alternative treatments [9]. In recent years, patients with liver cancer have been treated with Chinese herbal extracts. For example, it was investigated that *Crithmum maritimum* had significantly increased cellular respiration and decreased lactic fermentation in HCC cells. Inhibition of HCC was associated with this reduction of lactic fermentation [10]. A phenolics-rich fraction of leaves of *Pterospermum lanceifolium* fraction (PLE) was studied against hepatic cancer cell lines (HepG2) and an NDEA-induced HCC rat model system. The results showed that PLE induced reactive oxygen species (ROS) generation and chromatin condensation in the nucleus and altered the mitochondrial membrane potential (MMP) in HepG2 cell lines [11].

*Jacaranda mimosifolia* belongs to the dicot family Bignoniaceae and is present in tropical climates worldwide. The methanolic extract of *J. mimosifolia* showed the most potent anticancer activity against human lung cancer and prostate cancer cell lines [12]. Acetone, petroleum ether, and chloroform extracts prepared from *J. mimosifolia* were evaluated for their cytotoxic potential on the HCT-15 (Colorectal cancer cell line) cell line by MTT assay. Acetone crude extract displayed an IC_50_ value of 600 µg/ml, while petroleum ether and chloroform crude extracts possessed IC_50_ values of 800 µg/ml [13]. However, the detailed effects of *J. mimosifolia* on HCC and related molecular mechanisms are yet elusive.

With the advancement in the bioinformatics field, network pharmacology has improved significantly for drug discovery and design processes [14]. Network pharmacology is a powerful and well-defined method used to study the underlying mechanisms of traditional medicines and the interactions between target compounds and disease-associated proteins [15]. In this study, network pharmacology was conducted to establish the target-disease network to investigate the core targets and biological functions, pathways, and mechanisms of *J. mimosifolia* in treating HCC. Cell-based experiments and in vivo studies were conducted to explore the anti-cancer effect of *J. mimosifolia* against hepatocellular carcinoma.

## Materials and methods

### Reagents

High glucose Dulbecco’s Modified Eagle Medium (DMEM) (Gibco, Cat #12100061) and fetal bovine serum (FBS) (Gibco, Cat # 10500064) were purchased from Life Technologies Limited Paisley, PA49RF, UK. Dimethylsulfoxide (DMSO) (Sigma-Aldrich, Cat # 34943) was purchased from Sigma-Aldrich, St. Louis, MO, USA.

### Plant collection and extract preparation

Fresh *J. mimosifolia* leaves were identified and collected from the botanical garden of the University of the Punjab, and verified by Dr. Abdul Rehman Khan Niazi, professor in the botany department at the University of the Punjab, Lahore, Pakistan. The *J. mimosifolia* was assigned the voucher number LAH#060919. Leaves of *J. mimosifolia* were washed with water and then 70% ethanol, air-dried for 3 weeks, ground, and soaked in absolute methanol (1:3) for 4 days with periodic stirring. The resulting solution was filtered and the residue was soaked again. This process was repeated thrice. The filtrate was evaporated, and dried leaves crust was obtained. A stock solution of *J. mimosifolia* for *in vitro* experiments was prepared by dissolving 40 mg of dried crust in 1 ml of DMSO.

### *In vitro* and *in vivo* experiments

#### Cell culture.

The Huh-7.5 and Vero cell lines were obtained from the Nutraceutical and Microbial Biotechnology lab, CEMB. Both cell lines were cultured in DMEM supplemented with 10% FBS and 100 U/ml penicillin-streptomycin at 37°C in a humidified 5% CO_2_ incubator.

#### Cytotoxicity assay.

The cytotoxic potential of the methanolic extract of *J. mimosifolia* leaves was determined against the Huh-7.5 and Vero cell lines at doses of 3.12 µg/ml, 6.25 µg/ml, 12.5 µg/ml, 25 µg/ml, 50 µg/ml, 100 µg/ml, and 200 µg/ml after 24 h of treatment. Huh-7.5 and Vero cells were seeded at a density of 3.5 x 10^5^ cells/well for the MTT assay in a 96-well plate. Two wavelengths, i.e., test wavelength of 570 nm and the reference wavelength of 620 nm, were used for taking readings using a spectrophotometer.

*Percent cell viability = (sample 570nm- 620nm/control 570nm-620nm)x100*

#### Wound healing assay.

10 x 10^5^ Huh-7.5 cells were seeded into a 6-well plate and grown until 80% confluent cell monolayer. A linear wound was generated in the monolayer with a sterile 10 μl plastic pipette tip. The detached cells were removed by washing with phosphate buffer saline (PBS), and images of scratches were photographed at 0 h. A control (DMSO with media) and three *J. mimosifolia* extract doses of 50 μg/ml, 100 μg/ml, and 200 μg/ml were added, and cells were incubated at 37°C with 5% CO2. Images of the scratched areas were photographed to estimate the relative migration of cells after 24 h and 48 h, and were measured by ImageJ.

*Scratch closure (%) = (width of the scratch at 0h – width of scratch at 24h/ width of the scratch at 0h) x 100*

#### DNA fragmentation assay.

Huh-7.5 cells with a density of 10 x 10^5^ were seeded into a 6-well plate. After cell attachment, 100 μg/ml and 200 μg/ml doses of *J. mimosifolia* extract were added. DMSO with media was used as a negative control. After incubation for 24 h, cells were harvested by centrifugation and sequentially mixed with 100 μl of DMSO. An equal volume (100 μl) of 2% SDS with Tris-EDTA was added, and the resulting solution was centrifuged at 12000 rpm at 4°C for 25 minutes. 40 μl of supernatant from the control and each sample was loaded on agarose gel (1.5%). The gel was viewed under a UV transilluminator for clear results.

#### Hemolytic assay.

Human blood from a known healthy donor was obtained and centrifuged for five minutes at 500 x g. Plasma was aspirated gently using a micropipette. The initial mark of plasma was added to a 150 mM NaCl solution. The tube was covered and gently inverted a few times, centrifuged at 500 x g for 5 minutes, and the supernatant was removed. The same process was repeated twice to wash the blood cells. The tube was then filled to a marked level with PBS (pH 7.4), centrifuged at 500 x g for 5 minutes, and the supernatant was aspirated. The same process was repeated one more time. The tube was again filled with PBS (pH 7.4) at a marked level and gently inverted for mixing. 1 ml of erythrocytes was mixed with 49 ml of PBS for a 1:50 dilution. The erythrocytes were then treated with 10 μl of *J. mimosifolia*. 1% Triton X-100 was used as a positive control and PBS as a negative control. The plate was incubated at 37 °C for one hour followed by centrifugation at 25 °C for 5 min at 500 x g to make the pellet intact. 100 μl of supernatant was transferred from each well into a clear, flat-bottomed 96-well plate. The absorbance of supernatants was measured at 470 nm with a plate reader. The results were expressed as a percentage of hemolysis by the equation:

*% hemolysis = (Treatment absorbance−negative control absorbance)/ (positive control absorbance − negative control absorbance) × 100.*

#### RNA isolation, cDNA synthesis, and real-time PCR.

Total RNA was extracted from Huh-7.5 cells after 24 h incubation with 100 μg/ml and 200 μg/ml of *J. mimosifolia*, and DMSO with media (Control) using TRIzol reagent (ambion, USA) according to the manufacturer’s instructions. RNA was quantified by measuring optical density at 260 nm and 280 nm by nanodrop (NanoDrop-ND-1000). Reverse transcription was performed using RevertAid First Strand cDNA Synthesis Kit (Invitrogen Bioservices India Pvt. Ltd). cDNA was diluted 5 times, and Real-time PCR was performed with a Thermo Scientific PikoReal 96 Real-Time PCR System using Thermo Scientific Maxima SYBR Green/Fluorescein qPCR Master Mix (2X). The primers were designed and synthesized by Eurofins genomics (Germany) as follows: *β-actin*: 5′ - AGAGCTACGAGCTGCCTGAC-3’ (forward) and 5′ - AGCACTGTGTTGGCGTACAG-3’ (reverse); *p53*: 5′ -AAGGAAATTTGCGTGTGGAG −3’ (forward) and 5′ - CCAGTGTGATGATGGTGAGG −3’ (reverse); *Bax*: 5′ - ACCAAGAAGCTGAGCGAGTG-3’ (forward) and 5′ - AAGTAGAAAAGGGCGACAACC-3’ (reverse); *AFP*: 5′ -TGTCCCTCCTGCATTCTCTG-3’ (forward) and 5′ - ACCAAGCAGTACGTTCTCCA-3’ (reverse); *GPC3*: 5′ -TACTGCTCTTACTGCCAGGG-3’ (forward) and 5′ - TTGGCAGCATTTCTCCAACAG-3’ (reverse). Relative quantification of the mRNA levels was performed using the comparative Ct method by keeping *β-actin* as normalization control, and fold change was calculated with respect to the control group.

#### Mouse model.

All Swiss albino male mice (n = 20) were maintained at 23.0 ± 1 °C and relative humidity (60–70%) on a 12 h light/dark cycle with free access to food and water. After one week of adaptive feeding, animals were randomly divided into four experimental groups (n = 5 per group): normal control (NC), disease model (DM), and two *J. mimosifolia* treated groups. NC was intraperitoneally injected with olive oil, while remaining groups were intraperitoneally injected with CCl_4_ dissolved in olive oil (20% v/v, 1 ml/kg) thrice a week for consecutive six weeks. The NC and DM received normal saline, while *J. mimosifolia* treated groups received 100 mg/kg and 200 mg/kg separately thrice for consecutive four weeks by oral administration. At the end of the experiment, mice were euthanized using a two-step protocol. First, they were anesthetized using the isoflurane open-drop method, which produces rapid and smooth anesthesia with minimal distress and pain. Once they were fully anesthetized and unresponsive, cardiac puncture was performed as a secondary method that ensures irreversible cessation of circulation to confirm death. All efforts were made to minimize mice’s suffering throughout the procedure. Blood was collected from the heart, and serum was tested for liver biochemical markers. The liver was excised and kept in a 10% formalin solution for histological examination. The pathological status of the liver was confirmed with H&E staining and pathological examination.

## *In* *silico* studies

### Network pharmacology

#### Screening of active compounds of *J. mimosifolia.*

*J. mimosifolia* compounds were obtained from published literature. All of the compound’s canonical SMILES were retrieved from the PubChem database and were used to analyze the Lipinski rule of five and pharmacokinetic parameter including absorption, distribution, metabolism, and excretion (ADME) of compounds using SwissADME (http://www.swissadme.ch/) and admetSAR (https://lmmd.ecust.edu.cn/admetsar2). Subsequently, the compounds that met the criteria of ADME analysis and Lipinski rule were subjected to further evaluation.

#### Screening of potential targets for *J. mimosifolia* compounds.

Putative targets of selected compounds were found by providing their canonical SMILES on the SwissTargetPrediction online tool (http://www.swisstargetprediction.ch/). DisGeNET (https://www.disgenet.org/search) and GeneCards (http://www.genecards.org/) were used to obtain disease genes of HCC using the keyword “HCC”. A Venn diagram showing common targets of disease and compounds was constructed.

#### Gene function annotation.

Functional enrichment DAVID database (https://david.ncifcrf.gov/) was used for comprehensive annotation of genes. Gene ontology analysis provides information about MF (Molecular function), CC (Cellular component), and BP (Biological process). KEGG pathway analysis was also performed. “*Homo sapiens*” was selected as the model organism and only those pathways were selected whose probability score was less than 0.05. Results were visualized using Hiplot (https://hiplot-academic.com/).

### Network Pharmacology analysis

#### PPI interactions and prediction of Hub genes.

The STRING database (https://string-db.org/) evaluated the interaction between HCC targets. A list of common targets was pasted in the STRING database by selecting protein by name as “Multiple proteins” and species as “*Homo sapiens*”. Protein-protein interaction network was visualized using the Cytoscape. The CytoHubba plugin was used to find the hub genes; hub genes are those genes that are highly interactive. They were selected based on having a degree of ≥ 10 in the Cytoscape network.

#### Compound-target network construction.

Compound-Target network was created by Cytoscape (version 3.9.0) (https://cytoscape.org/release_notes_3_9_0.html). Active compounds of *J. mimosifolia* and common targets were used to find most interacted genes with compounds (using degree method). The compounds and targets are represented by the nodes, while the interactions are shown by the edges in the network. The “Degree” is used to filter the network as it represents the number of connected nodes for a specific node.

#### Construction of compound-target-pathway network.

Based on KEGG pathway analysis, the pathways were determined using DAVID to create a compound-target- pathway network in Cytoscape.

#### Molecular docking.

Compounds were docked with hub genes (HCC proteins) using Glide in Schrodinger software. HCC proteins were obtained from AlphaFold in PDB format. The following criteria were used to select the proteins for docking: (1) protein should be complete (2) should have smaller resolution values (below 2 Å) (3) should be human proteins. Docking complexes with the lowest docking score were selected. Docked complexes were converted to PDB in PyMOL and visualized with Discovery Studio.

### Statistical analysis

All the statistical analyses were performed with GraphPad Prism 9. Tukey’s multiple comparison was used for one-way ANOVA and the significance level was set at p ≤ 0.05 for all the *in vitro* experiments and *in vivo* studies.

## Results

### Cell viability analysis of cancerous and normal cell lines

**Fig 1:**
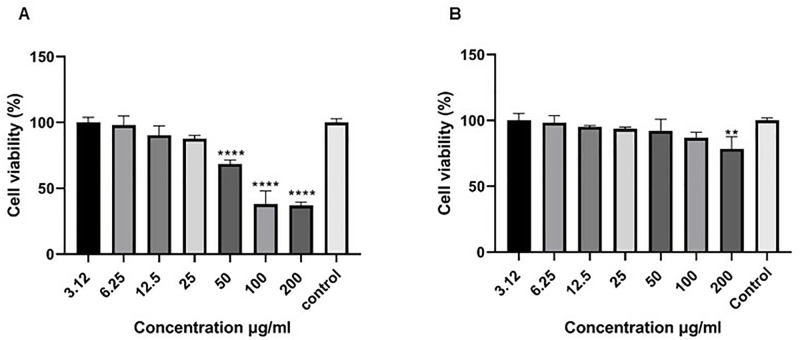
Cytotoxic effect of *J. mimosifolia* on cell viability. (A) Dose-dependent effect of *J. mimosifolia* on Huh-7.5 cell viability. (B) Dose-dependent effect of *J. mimosifolia* on Vero cell viability. Each error bar expresses mean ± SD; n = 3. **p < 0.01 and ****p < 0.0001 compared with control.

At 24 h treatment period, *J. mimosifolia* extract reduced (P < 0.0001) Huh-7.5 cell viability to 68.6%, 38.1%, and 36.8% at 50 μg/ml, 100 μg/ml, and 200 μg/ml doses, respectively, as compared to untreated Huh-7.5 cells (Control) (Fig 1A). Thus, the cell viability data suggest that *J. mimosifolia* treatment significantly reduces Huh-7.5 cell growth in a dose-dependent manner, indicating its ability to impair proliferation potential. *J. mimosifolia* extract showed low toxicity against Vero cells, and the percent cell viability after 24 h was found to be 92.1%, 86.8%, and 80.4% at 50 μg/ml, 100 μg/ml, and 200 μg/ml, respectively (Fig 1B). However, *J. mimosifolia* extract exhibited 80% survival on normal cells compared to Huh-7.5 cells (36%) at the highest dose of 200 μg/ml, indicating that its cytotoxic activity is selective for the cancer cell line.

### Cell migration analysis of cancerous cells

**Fig 2:**
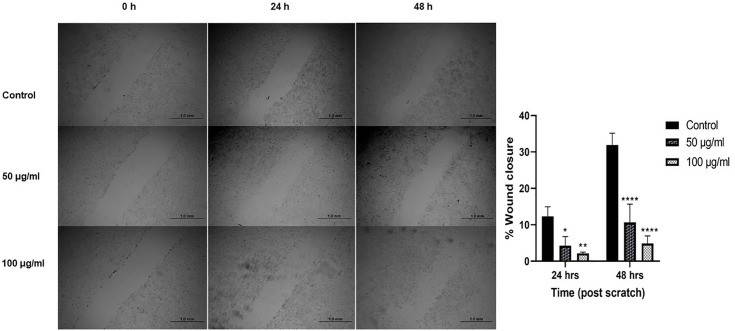
Effect of *J. mimosifolia* on wound healing 0, 24, 48 h after wound creation. Each error bar expresses mean ± SD; n = 3.*p < 0.05, **p < 0.01, and ****p < 0.0001 compared with control.

The wound healing method was used to assess the cell migration of Huh-7.5 cells after incubation with different concentrations (50, 100, and 200 μg/ml) of extract for 24 h and 48 h. It was found that 50 μg/ml and 100 μg/ml *J. mimosifolia* extract induced dose-dependent inhibition of cell migration in Huh-7.5 compared to untreated Huh-7.5 cells (Control) which showed a natural rate of cell migration. Huh-7.5 cells treated with a dose of 200 μg/ml became detached after 24 h of treatment from the surface of the plate. Huh-7.5 cells treated with 50 μg/ml and 100 μg/ml doses for 48 h showed dose-dependent migratory inhibition with P < 0.0001. These results indicate that *J. mimosifolia* inhibits the proliferative and migratory potential of Huh-7.5 cells. The detached 200 μg/ml treated Huh-7.5 cells became clustered and floated in the media after 48 h. This detachment could indicate cell death or a significant disruption in the adhesion and migration of the HCC cells (Fig 2).

### Analysis of cancerous cells with damaged DNA

**Fig 3:**
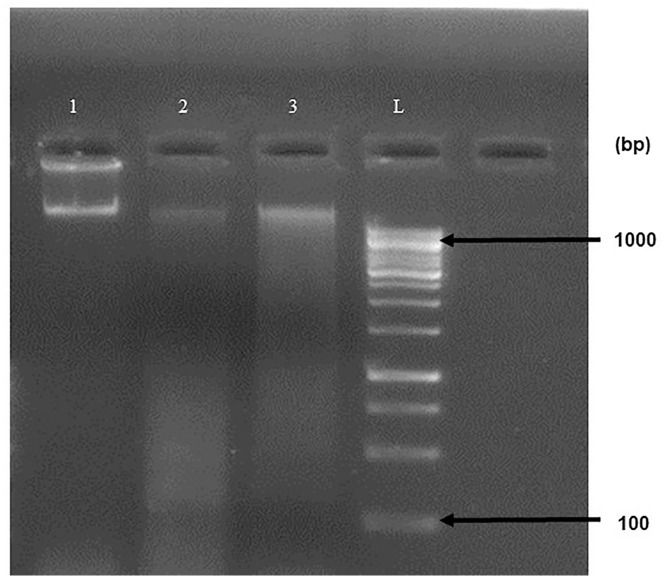
DNA fragmentation assay for the DNA extracted from control and *J. mimosifolia*-treated Huh-7.5 cells. Lane 1: Control; Lane 2: DNA extracted from 100 μg/ml *J. mimosifolia-*treated Huh-7.5 cells; Lane 3: DNA extracted from 200 μg/ml *J. mimosifolia-*treated Huh-7.5 cells; Lane L: DNA ladder.

DNA fragmentation is a hallmark feature of apoptosis. Huh-7.5 cells treated with 100 μg/ml and 200 μg/ml of *J. mimosifolia* extract induced a smear pattern of DNA fragmentation, while untreated Huh-7.5 cells (control) yielded a clear band of intact DNA after 24 h of treatment (Fig 3).

### Determination of hemolytic activity of *J. mimosifolia* extract

**Fig 4:**
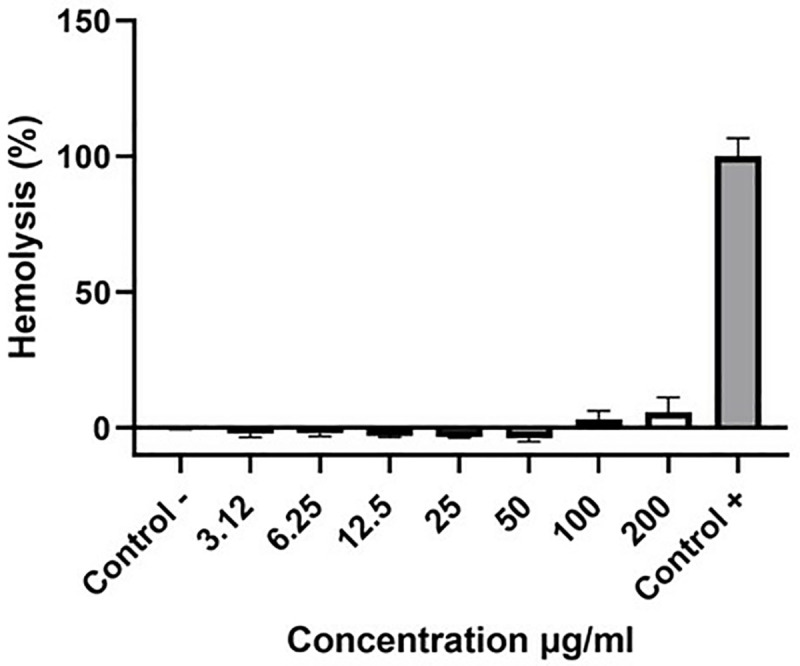
Hemolytic activity of *J. mimosifolia* against human erythrocytes. Each error bar expresses mean ± SD; n = 3.

Not a single tested dose caused any significant hemolysis to erythrocytes (P > 0.05) when compared to negative control. The percentage of hemolysis was negligible in the negative control while hemolysis was 100% in positive control. Only two doses, i.e., 100 μg/ml and 200 μg/ml showed the highest hemolysis which was only 3.2% and 5.4% respectively, and was not significant compared to negative control (Fig 4).The percentage of hemolysis did not exceed 10% with all doses indicating their non-cytotoxicity to healthy erythrocytes.

### Gene expression analysis

**Fig 5:**
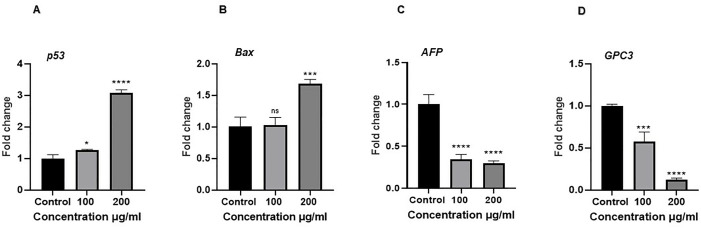
Gene expression analysis of HCC markers. **(A)** *p53* **(B)** *Bax* **(C)** *AFP* **(D)** *GPC3*. Each error bar expresses mean ± SD; n = 3. ns = p > 0.05 (not significant),*p < 0.05,***p < 0.001, and ****p < 0.0001 compared with control.

The expression level of *p53* (P < 0.0001) and *Bax* (P < 0.001) was increased, and that of *AFP and GPC3* was decreased (P < 0.0001) in *J. mimosifolia-*treated Huh-7.5 cells as compared to control cells (Fig 5).

### Effects of *J. mimosifolia* on mice with hepatic injury

**Fig 6:**
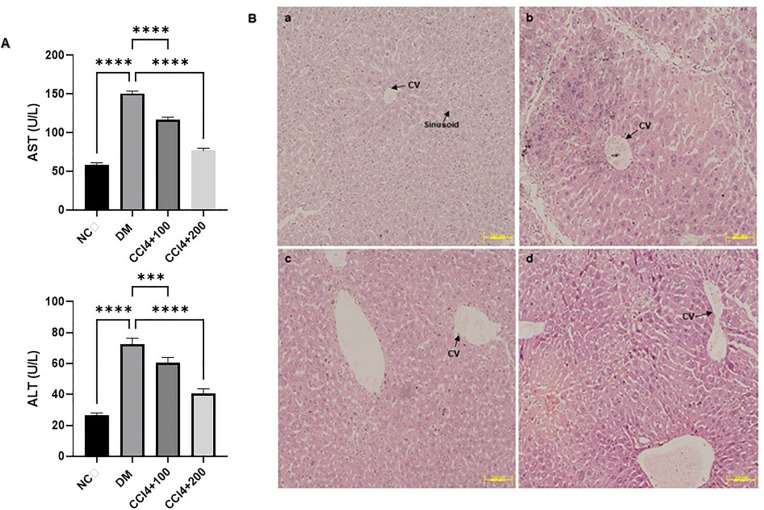
(A) Levels of liver biochemical markers (ALT and AST) in mice serum. NC = Normal Control, DM = Disease Model, CCl4 + 100 mg/kg = Disease model treated with 100 mg/kg *J. mimosifolia*, CCl4 + 200 mg/kg = Disease model treated with 200 mg/kg *J. mimosifolia.* Each error bar represents the mean ± SD; n = 5. ****p < 0.0001 compared with the control. **(B)** Histopathological analysis of mice liver (100X). **(a)** Normal Control group without CCl_4_ treatment shows normal central vein (CV), well-packed hepatocytes with intact nucleus and normal sinusoids; **(b)** Disease Model group treated with CCl_4_ only exhibits congested central vein (CV), ballooning degeneration of hepatocytes, and prominent signs of inflammation; **(c)** CCl_4_ + 100 mg/kg *J. mimosifolia* treated group demonstrates mild congested central vein (CV), slight sinusoidal dilations (SD) and mild inflammation; **(d)** CCl_4_ + 200 mg/kg *J. mimosifolia* treated group shows normal central vein (CV), normal sinusoids (NS), and well-preserved hepatocytes.

The levels of biochemical markers AST and ALT were significantly increased (p < 0.0001) in the disease model group (DM) compared to the normal control (NC) group, which did not receive CCl_4_ treatment. In contrast, elevated levels of these enzymes were restored (p < 0.0001) in the treated groups that received *J. mimosifolia* extract treatment at 200 mg/kg, indicating recovery from hepatic injury (Fig 6A). The hepatoprotective effect of *J. mimosifolia* on CCl_4_-induced liver damage was further confirmed by histopathological examination. The liver samples from the DM group showed disruption of normal hepatic architecture, particularly in centrilobular areas. Hepatocytes exhibited degenerative changes, including cytoplasmic vacuolization and ballooning, accompanied by sinusoidal dilatation and congestion. Marked inflammatory cell infiltration and necrosis were present, indicating hepatocellular injury. However, preserved hepatic architecture was observed in the groups administered with *J. mimosifolia*. 100 mg/kg group exhibited slight sinusoidal dilatation, minimal hepatocyte degeneration, and mild necrosis and inflammation whereas the 200 mg/kg group showed normal sinusoids and well-preserved hepatocytes with very mild inflammation, indicating enhanced restoration of normal liver histology (Fig 6B).

### Network pharmacology

#### Screening of targets for compounds of *J. mimosifolia.*

**Fig 7:**
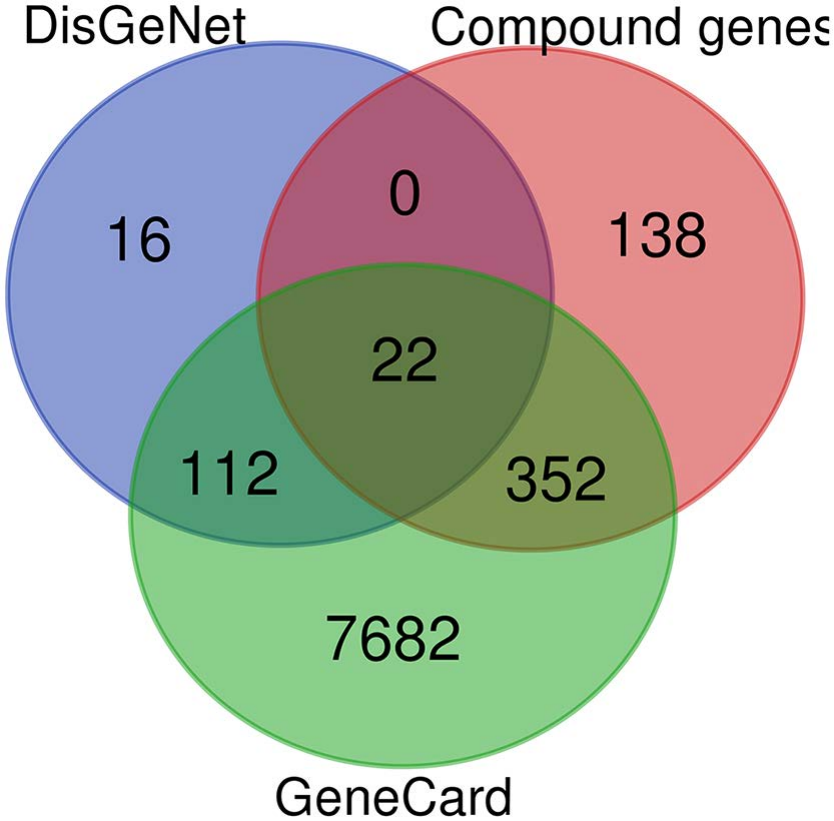
Venn diagram showing 22 common compound-disease targets.

About 35 *J. mimosifolia* compounds were obtained from published literature using Google Scholar and PubMed. After performing the Lipinski rule of five and ADME analysis on these 35 compounds, only 12 were selected as effective components (Tables 1 and 2). 512 targets of 12 active compounds of *J. mimosifolia* were retrieved through the SwissTargetPrediction database. The GeneCard and DisGeNet databases identified 8168 and 150 possible targets of HCC, respectively. Potential mapping between active compounds’ targets and HCC targets found 22 common targets (Fig 7 and S1 Table), which were considered potential targets of *J. mimosifolia* against HCC.

**Table 1:** Selected *J. mimosifolia* compounds with putative drug like properties.

Sr no.	Compounds	MW(<500 g/mol)	ClogP < 5	HBA < 10	HBD < 5	RB < 10
1.	**Apigenin**	270.239	2.3357	5	3	1
2.	**Caffeic Acid**	180.159	0.7825	4	3	2
3.	**Gallic acid**	170.12	0.1076	5	4	1
4.	**Kaempferol**	286.238	1.8359	6	4	1
5.	**Trans-cinnamic acid**	148.161	1.4739	2	1	2
6.	**4 Hydroxyphenylacetic Acid**	152.149	0.7971	3	2	2
7.	**Benzoic acid**	122.123	1.1447	2	1	1
8.	**Citronellyl Propionate**	212.332	4.2884	2	0	8
9.	**Flavonol Glycoside**	400.382	1.0359	8	4	4
10.	**4 hydroxypentan-2-one**	102.132	0.2656	2	1	2
11.	**Lapachol**	242.273	3.1688	3	1	2
12.	**Hydroquinone**	110.112	0.9682	2	2	0

**Abbreviations:** MW, molecular weight; HBD, the number of hydrogen bond donors; HBA, the number of hydrogen bond acceptors; RB, the number of rotatable bonds; ClogP, lipophilicity. No compound violated the Lipinski rule.

**Table 2:** ADME Analysis of Twelve screened compounds of *J. mimosifolia.*

Compounds	ADME analysis							
	GI absorption	BBB permeant	Pgp substrate	CYP1A2 inhibitor	CYP2C19 inhibitor	CYP2C9 inhibitor	CYP2D6 inhibitor	CYP3A4 inhibitor
Apigenin	High	Yes	No	Yes	Yes	Yes	Yes	Yes
Caffeic acid	High	Yes	No	Yes	Yes	Yes	No	Yes
Gallic acid	High	Yes	No	No	No	No	No	Yes
Kaempferol	High	Yes	Yes	Yes	Yes	Yes	Yes	Yes
Trans-Cinnamic acid	High	Yes	No	No	No	No	No	No
4-Hydroxyphenylacetic acid	High	Yes	No	No	No	No	No	No
Benzoic acid	High	Yes	No	No	No	No	No	No
Citronellyl propionate	High	Yes	No	No	No	No	No	No
Flavonol glycoside	High	No	Yes	No	No	No	No	No
4-Hydroxypentan-2-One	High	Yes	No	No	No	No	No	No
Lapachol	High	Yes	Yes	Yes	Yes	Yes	No	No
Hydroquinone	High	Yes	No	No	No	No	No	Yes

#### PPI network and compounds target network construction.

**Fig 8:**
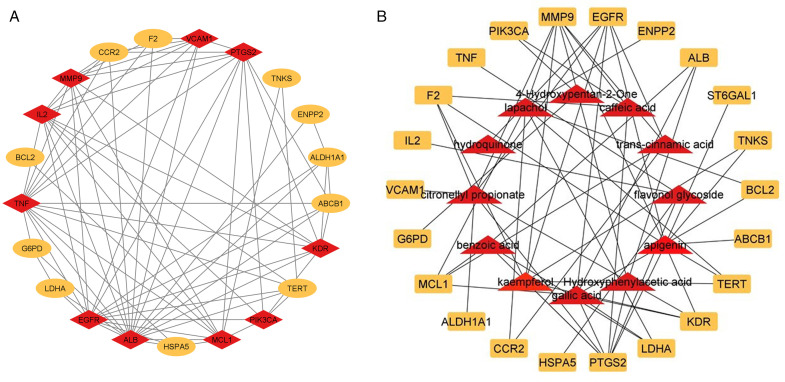
Linkage of target genes and target compounds. (A) PPI network analysis of 22 targets. (B) Compound-Target network where red nodes represent active compounds and light-orange nodes represent common targets.

Twenty-one nodes and 76 edges were found in the PPI network using Cytoscape analysis (Fig 8A). One gene, ST6GAL1, did not show any interaction with other target genes. Ten hub genes were identified using the CytoHubba plugin. A network was created between common targets and active compounds to study their interactions, revealing that the compound-target network consisted of 34 nodes and 62 edges (Fig 8B). The degree scores of active compounds with their corresponding targets were also computed (Table 3).

**Table 3:** Twelve active compounds of *J. mimosifolia* with degree scores.

Sr no.	Compounds	Degree
**1.**	Apigenin	9
**2.**	Kaempferol	8
**3.**	Citronellyl propionate	7
**4.**	Lapachol	7
**5.**	Gallic acid	7
**6.**	Caffeic acid	4
**7.**	Flavonol glycoside	4
**8.**	Benzoic acid	4
**9.**	Trans-cinnamic acid	4
**10.**	4-Hydroxyphenylacetic acid	3
**11.**	4-Hydroxypentan-2-One	2
**12.**	Hydroquinone	1

#### Compound-target-Pathway network analysis.

**Fig 9:**
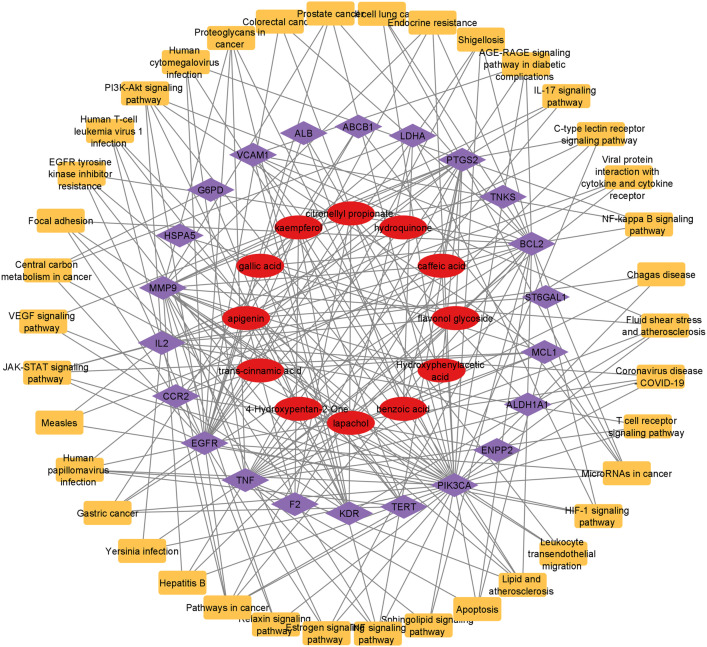
Compound-target-pathway network. Red nodes represent active compounds, purple nodes represent common targets, and light-orange nodes represent pathways.

The compound-target-pathway network was created using Cytoscape by the selection of potential pathways, active compounds, and disease targets. The network contained 72 nodes and 216 edges (Fig 9). The targets of *J. mimosifolia* showed coordination with diverse pathways.

#### Go and KEGG analysis.

**Fig 10:**
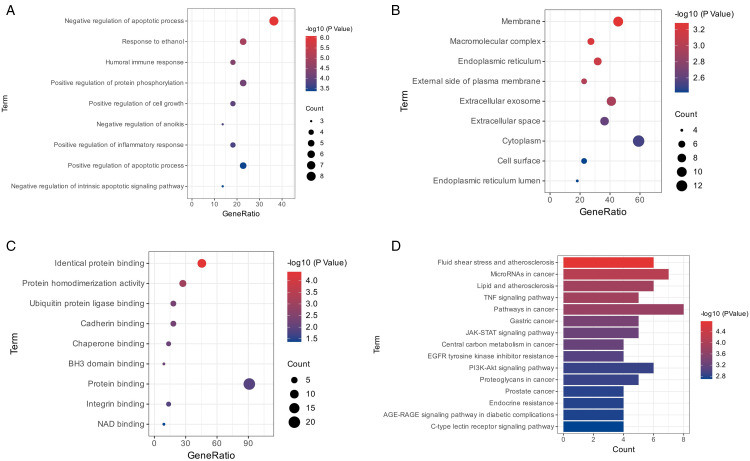
Bubble map for GO analysis and bar graph for KEGG analysis. **(A)** Biological Process (BP); **(B)** Cellular Component (CC); **(C)** Molecular Function (MF); **(D)** KEGG analysis.

The gene ontology and molecular mechanisms of *J. mimosifolia* associated with HCC were predicted using GO and KEGG analysis, respectively. After applying a cutoff value of P ≤ 0.05, 71 biological processes (BP), 14 cellular components (CC), 9 molecular functions (MF), and 38 pathways were found. BP included regulation of apoptotic process, protein phosphorylation, and inflammatory response (Fig 10A); CC involved membrane, extracellular exosomes, and endoplasmic reticulum (Fig 10B); and MF consisted of cadherin binding, ubiquitin protein ligase binding, and chaperone binding (Fig 10C). KEGG pathways included fluid shear stress and atherosclerosis, MicroRNAs in cancer, PI3K-Akt signaling pathway, TNF signaling pathway, and JAK-STAT signaling pathway (Fig 10D).

#### Molecular docking analysis.

**Fig 11:**
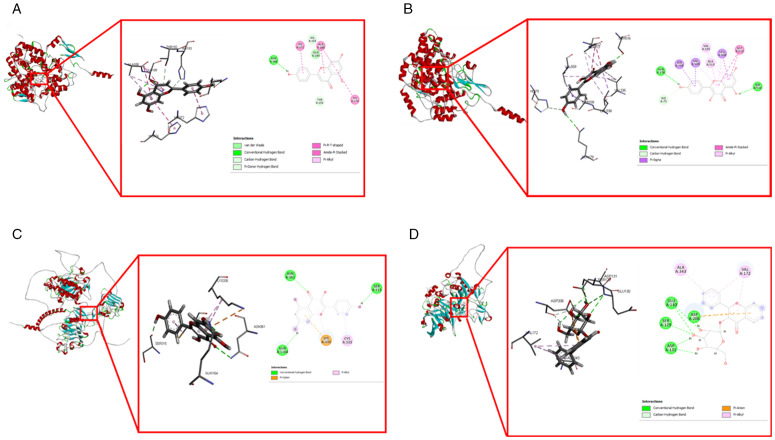
Docking results of compounds with their targets. **(A)** Docking complex of PTGS2 with Apigenin. **(B)** Docking complex of PTGS2 with Kaempferol. **(C)** Docking complex of EGFR with Kaempferol. **(D)** Docking complex of MMP9 with Flavonol glycoside.

Docking analysis predicted the amount of energy released during the interactions between compounds and the binding sites on targets. Five target genes that are overexpressed in HCC and had the highest degrees were selected for molecular docking, i.e., *EGFR*, *TNF*, *PTGS2*, *MMP9*, and *MCL1*. So, all 12 compounds docked with five potential targets of HCC. The binding score ranged from −3.4 to −8.7 kcal mol ⁻ ¹ (Table 4). Apigenin and kaempferol showed the lowest binding energies of −8.7 and −7.4 kcal mol ⁻ ¹ with PTGS2 protein, respectively. Kaempferol also showed a binding energy of −7.4 kcal mol ⁻ ¹ for EGFR. Flavonol glycoside showed the lowest binding energy of −8.4 kcal mol ⁻ ¹ with the MMP9 (Fig 11).

**Table 4:** Docking results show the binding energies of potential targets with *J. mimosifolia* compounds.

Compounds	Binding Energy (kcal mol ⁻ ¹)				
	EGFR	TNF	PTGS2	MMP9	MCL1
**Apigenin**	−7.2	−5.9	−8.7	−7.7	−6.7
**Kaempferol**	−7.4	−6.1	−7.4	−7.0	−6.4
**Citronellyl propionate**	−4.9	−3.8	−4.7	−4.5	−4.6
**Lapachol**	−6.7	−4.1	−6.8	−6.3	−6.0
**Gallic acid**	−5.5	−5.6	−6.7	−5.8	−5.5
**Caffeic acid**	−7.2	−5.0	−6.3	−5.9	−5.6
**Flavonol glycoside**	−5.7	−6.7	−7.0	−8.4	−6.6
**Benzoic acid**	−5.2	−4.7	−5.8	−5.0	−4.8
**Trans-cinnamic acid**	−5.7	−4.3	−5.9	−5.4	−5.3
**4-Hydroxyphenylacetic acid**	−5.4	−5.6	−6.0	−5.6	−5.3
**4-Hydroxypentan-2-One**	−4.1	−3.4	−4.5	−3.8-	−3.5
**Hydroquinone**	−4.6	−4.2	−5.4	−5.1	−4.3

## Discussion

Liver cancer is the second leading cause of mortality and the seventh most common disease in the world [16]. In Pakistan, 10.7% of all cancer cases are HCC malignancies, which are the most prevalent kind of cancer in adult males [17]. Although there are surgical and medical treatments available, surgery has limited therapeutic options, especially for patients with advanced HCC, and drugs pose serious side effects [18]. This study investigated the potential of using *J. mimosifolia*, an alternative source of plant-based compounds that may reduce the adverse effects of traditional medications and inhibit cancer proliferation.

Our results align with previous studies employing medicinal plants that target liver cancer cells *in vitro*. Cytotoxicity of *J. mimosifolia* was determined through cell viability assay against the Huh-7.5 cell line and significant dose-dependent anti-proliferative effects were observed (Fig 1A). Naz *et al*. also showed cytotoxic activity of the methanolic extract of *J. mimosifolia* against LU-1 and LnCaP cell lines [12].The cytotoxicity of *J. mimosifolia* extract was also assessed against Vero cells (a normal cell line), ensuring it selectively targets Huh-7.5 cells. Interestingly, the *J. mimosifolia* extract did not demonstrate significant toxicity against Vero cells (Fig 1B), suggesting that *J. mimosifolia* is more toxic to liver cancer cells (Huh-7.5). A previous study also showed that the ethanolic extract of Ajwa date pulp (ADP) significantly inhibited the growth of human liver cancer HepG2 cells while having little to no effect on normal Vero cells [19]. In our study, wound healing assay showed significant inhibition in the migration of *J. mimosifolia* treated Huh-7.5 cells in a dose- dependent manner as compared to the untreated Huh-7.5 cells after 24 h. A minimal migration rate indicates that *J. mimosifolia* inhibits the proliferative and migratory capabilities of Huh-7.5 cells (Fig 2). Similar anti-migratory effects were also observed by *Acacia modesta* and *Opuntia monocantha* against liver cancer cells in a wound-healing assay [20]. However, Huh-7.5 cells treated with 200 μg/ml dose of *J. mimosifolia* extract became detached from the surface after 24 h of treatment. Wang *et al*. in a previous study investigated the roles of cytotoxic benzophenanthridine alkaloids in biological processes related to cell migration and cytoskeleton dynamics. It was noted that tumor cells were detached from the surface at higher doses of 5000 nM, 12,000 nM, and 20,000 nM of sanguinarine, chelerythrine, and chelidonine respectively. These alkaloids influenced the microtubule network and caused microtubule depolymerization that eventually resulted in apoptosis, even though they did not significantly inhibit cell migration [21]. Based on the above study, it is suggested that the *J. mimosifolia* extract at a higher concentration of 200 ug/ml similarly affects the cytoskeletal network, potentially leading to microtubule disruption and apoptosis. However, further investigation is needed to determine whether this detachment is associated with microtubule depolymerization or other mechanisms contributing to apoptosis. In our study, treatment of Huh-7.5 cells with *J. mimosifolia* extract resulted in DNA fragmentation (Fig 3), as evidenced by a smear pattern on the gel rather than the classic DNA ladder. Although a ladder pattern is often produced by internucleosomal DNA cleavage during apoptosis, it is well known that apoptotic DNA fragmentation can alternatively appear as a smear. This variation can be affected by factors, including cell type, the nature of apoptotic stimuli, and the degree of DNA degradation. A study by Kumar *et al*. showed that treatment with *Moringa oleifera* leaf extract caused DNA fragmentation, which was seen as a smear on the gel, showing apoptosis in cancer cells [22]. The presence of a smear pattern may also indicate that the apoptotic cells have entered late apoptosis (secondary necrosis) due to a lack of phagocytosis [23]. All seven doses of *J. mimosifolia* extract were evaluated for their hemolytic ability. Not a single dose caused any damage to the membrane of erythrocytes in our study (Fig 4). While 1% Triton X-100 used as a positive control caused 100% erythrocyte lysis and PBS used as a negative control showed negligible hemolysis, these findings are similar to previous studies [24–27].

The expression levels of *p53* and *Bax* were significantly increased in *J. mimosifolia* treated Huh-7.5 cells compared to control Huh-7.5 cells while expression levels of *AFP* and *GPC3* were significantly reduced in *J. mimosifolia* treated Huh-7.5 cells as compared to control cells (Fig 5). *p53* is a phosphate protein found in the cell nucleus that regulates how a cell reacts to various types and levels of stress by triggering apoptosis, cell cycle arrest, senescence, DNA repair, cell metabolism, or autophagy [28,29]. Furthermore, it regulates several genes that are probably involved in apoptosis, including the gene for the pro-apoptotic protein *Bax*, which is associated with *Bcl*-2, and several genes that encode proteins involved in the generation of reactive oxygen species (ROS) [30–32]. If *p53* is mutated, DNA-damaged cells can evade apoptosis and become malignant cells [33]. The silencing of *p53* tumor suppressor gene is one of the most common abnormalities in a variety of malignancies, most notably HCC. Data from genome analysis revealed that 25–30% of the HCC patients have mutations in the *p53* gene while half of the HCC patients have dysfunctional *p53* signaling [34]. Elevated *p53* protein levels trigger apoptosis in tumor cells by blocking cell proliferation through numerous biological pathways and making HCC vulnerable to different anticancer drugs [35]. *Bax*, as an accelerator of apoptosis has reduced levels in HCC. According to studies, *Bax* overexpression increases apoptosis in ovarian cancer cell lines and sensitizes human head and stomach cancer cells to different chemotherapeutic drugs [36–38]. Zheng *et al*. reported that overexpression of *Bax* not only caused apoptosis but also caused HCC-9204 cells sensitive to Adriamycin-induced cell death [39]. Increased levels of *p53* and *Bax* in our study may indicate the role of *J. mimosifolia* extract in inducing cellular apoptosis.

*AFP* is the most commonly used tumor marker for HCC. It is an oncofetal glycoprotein that is naturally expressed in fetuses [40,41]. The complex genetic control of *AFP* has not yet been extensively characterized, although it is expressed in 60% to 80% of HCC cases [42]. *AFP* controls the proliferation of both normal and cancerous cells through a variety of processes, such as cytoplasmic signaling modulation and apoptotic regulation. It has been demonstrated that *AFP* protects HepG2 and HL-60 cells against apoptosis induced by various factors [43,44]. Hepatoma cells become more sensitive to tumor necrosis factor-related apoptosis-inducing ligand (TRAIL) when *AFP* is knocked down, which in turn triggers the caspase-3 signaling. Consequently, the chemotherapeutic efficacy of TRAIL may be increased by combining TRAIL therapy with *AFP* gene silencing [45]. Reduced levels of *AFP* by *J. mimosifolia* extract could demonstrate its role in inhibiting the pro-proliferative activity of *AFP*.

*GPC3* is a crucial molecular target in HCC and can serve as a therapeutic target for the treatment of HCC, as evidenced by the high expression of *GPC3* in 70–100% of cases [46]. A typical characteristic of HCC is the canonical Wnt signaling pathway, which is stimulated by *GPC3* to promote the development of HCC [47]. *GPC3* overexpression also affects cell proliferation by inhibiting the function of bone morphogenetic protein 7 (BMP-7) and fibroblast growth factor 2 (FGF2) [48]. Mechanistic studies revealed that *GPC3* is involved in the regulation of the tumor microenvironment and the dissemination of cancer. Heparan sulfate chain-mediated interaction with the HGF/Met pathway demonstrated that *GPC3* played a role in HCC cell migration and motility. *GPC3* gene-specific shRNA was used to knock down *GPC3*, which decreased HCC cell migration and motility [49]. Wu *et al.* found a clinical correlation between the expression of EMT-associated proteins, tumor vascular invasion and elevated levels of *GPC3* in HCC tumor tissues. Additionally, it was found that *GPC3* can regulate HCC cell EMT by triggering p-ERK1/2 signaling [50]. Reduced levels of *GPC3* induced by *J. mimosifolia* extract may indicate its role in preventing cancerous cells’ proliferation, migration, and metastasis.

Our results showed that injecting mice with CCl_4_ caused significant liver damage, reflected by an increase in AST and ALT serum levels. When liver cells are damaged, their functional state is altered; membrane permeability increases, allowing enzymes to leak into the extracellular environment [51,52]. The severity of CCl_4_-induced liver damage was significantly reduced by *J. mimosifolia* treatment. *J. mimosifolia* can stabilize liver cell membranes and prevent enzyme leakage, as shown by the recovery of treated mice to nearly normal enzyme levels (Fig 6A). The hepatoprotective effect of *J. mimosifolia* on CCl_4_-induced liver damage was further confirmed via histopathological examinations. The liver samples of mice administered with only CCl_4_ indicated damages such as necrotic hepatocytes and infiltration of inflammatory cells [53]. However, in the groups administered with *J. mimosifolia*, minimal necrosis and inflammation in the liver was observed compared to CCl_4_ group (Fig 6B). In a previous study, HCC-bearing rats were treated with *Dioscorea membranacea,* and cancer areas were significantly reduced compared to the untreated group [54].

Network pharmacology utilizes computational tools to identify and analyze interactions between compounds and disease genes for discovering drug candidates [55]. In our study, 12 compounds of *J. mimosifolia* were used against 22 HCC genes. GO analysis and KEGG pathways enrichment analysis were also performed. Only hub genes with the highest degree scores for KEGG pathways were selected for docking. *EGFR*, *TNF*, *PTGS2*, *MMP9*, and *MCL1* were selected and docked against 12 active compounds of *J. mimosifolia*.

The *EGFR* is increased in liver macrophages, where it serves a tumor-promoting function, in both human and mouse HCC models [56]. In liver biopsies from chronic HCC patients with and without cirrhosis, serum TNF- was discovered to be positively correlated with both inflammation and fibrosis [57]. Aggressive characteristics and a poor prognosis are correlated with upregulated *PTGS2* expression in HCC. Overexpression of *PTGS2* inhibited mitophagy, a process that contributes to mitochondrial dysfunction in HCC cells while protecting normal cells from accumulated mitochondrial damage [58]. By stabilizing *MMP9* mRNA, the RNA binding protein L23 may increase *MMP9* expression and, consequently, promote HCC metastasis [59]. *MCL-1* controls intrinsic apoptosis induction at the mitochondrial level and is frequently overexpressed in human cancer [60].

BP GO revealed that targets showed a notable response in the regulation of the apoptotic process and protein phosphorylation (Fig 10A). In HCC, several physiological pro-apoptotic molecules are either downregulated or rendered inactive. However, hepatocarcinogenesis, regardless of the presence of inflammation, can arise from increased apoptosis during spontaneous tumor development [61]. The phosphoproteins located in the cell nucleus may be linked to the processes of hepatic oncogenesis, as implied by the subcellular distributions of HCC phosphoproteins. Notably, the discovery that 29 dysregulated phosphoproteins are associated with mitochondria, which play a critical role in cell metabolism, survival, and apoptosis, suggests that mitochondria could significantly contribute to hepatic oncogenesis [62].

CC GO analysis showed that targets were primarily localized to the plasma membrane and endoplasmic reticulum (Fig 10B). The development of HCC depends primarily on cytoplasmic membrane proteins, which are extensively expressed in hepatic tumor tissues [63]. Endoplasmic reticulum stress driven by cellular disruptions like lipotoxicity is implicated in developing and progressing several hepatic diseases, including liver cancer [64].

MF GO analysis showed that targets were significantly involved in ubiquitin-protein ligase and cadherin binding (Fig 10C). Through a cascade of enzymatic reactions triggered by the ubiquitin-proteasome system (UPS), ubiquitin attaches to the targeted protein sequentially. The development of HCC is significantly correlated with proteins that serve as UPS enzymes, and the unchecked ubiquitination of essential proteins transduces signaling within HCC cells [65]. Disease pathogenesis, such as tumor growth and metastasis, is frequently associated with the dysregulation of cadherin (cell adhesion molecule) expression. Genetic polymorphisms and alternative splice isoforms of the *CDH17* have been found in HCC and are associated with a higher risk of developing HCC [66].

In our study, KEGG pathway analysis revealed that many of the targets are involved in various cellular pathways. Targets were found to be enriched in the TNF signaling pathway, the PI3K/AKT signaling pathway, and the JAK/STAT pathway (Fig 10D). Tumor necrosis factor (TNF) is a cytokine that promotes inflammation, and its expression is dramatically higher in HCC than in healthy liver tissue [67]. One of the most significant intracellular pathways, the PI3K/AKT signaling pathway controls cellular metabolism, cell proliferation, differentiation, and survival. The PI3K/AKT signaling pathway, which is active in 30% to 50% of HCC, plays a significant role in the disease [68]. HCC initiation and progression have been linked to abnormal JAK/STAT pathway activation [69].

Twelve compounds were docked against hub genes. Apigenin was successfully docked against PTGS2, while Kaempferol was successfully docked against both PTGS2 and EGFR. Additionally, flavonol glycoside was docked against MMP9, resulting in the lowest docking score (Fig 11 and Table 4). The remaining compounds against hub genes also exhibited high docking scores. The docking results are consistent with previously reported *in vitro* and *in silico* approaches [70,71].

## Conclusion

This study demonstrated that *J. mimosifolia* could efficiently suppress Huh-7.5 cellular proliferation, migration, and induce cytotoxicity. In addition, *J. mimosifolia* has the potential to reverse hepatic injury in mice. The pharmacological mechanisms by which *J. mimosifolia* inhibited HCC were investigated through network pharmacology, and potential phytochemicals of *J. mimosifolia* against HCC were found by molecular docking. To summarize, the data from this study provide initial evidence that *J. mimosifolia* extract may be considered a potential natural drug candidate for HCC. However, further research is necessary to better understand the therapeutic potential of this plant.

## Supporting information

S1 Table Potential common HCC proteins targeted by of *J. mimosifolia.* (DOCX)

S1 File Raw image. (PDF)
