# Supplementary material for: Anti-hepatocellular carcinoma activity of Jacaranda mimosifolia through experimental validation and network pharmacology
Source: PLoS One. 2026 Apr 3;21(4):e0346325. doi: 10.1371/journal.pone.0346325 (PMC13048444; doi:10.1371/journal.pone.0346325)
Supplement: S1 Table — (DOCX) [file pone.0346325.s001.docx]

| **Sr.no.** | **UniProt ID** | **Protein name** | **Gene name** |
| --- | --- | --- | --- |
| 1. | P00533 | Epidermal growth factor receptor | EGFR |
| 2. | P11413 | Glucose-6-phosphate dehydrogenase | G6PD |
| 3. | P02768 | Albumin | ALB |
| 4. | P08183 | ATP binding cassette subfamily B member 1 | ABCB1 |
| 5. | P00338 | Lactate dehydrogenase A | LDHA |
| 6. | O95271 | Tankyrase | TNKS |
| 7. | P01375 | Tumor necrosis factor | TNF |
| 8. | P35354 | Prostaglandin-endoperoxide synthase 2 | PTGS2 |
| 9. | P10415 | BCL2 apoptosis regulator | BCL2 |
| 10. | P15907 | ST6 beta-galactoside alpha-2,6-sialyltransferase1 | ST6GAL1 |
| 11. | Q07820 | MCL1 apoptosis regulator, BCL2familymember | MCL1 |
| 12. | P42336 | phosphatidylinositol-4,5-bisphosphate 3-kinase  catalytic subunit alpha | PIK3CA |
| 13. | P00352 | Aldehyde dehydrogenase 1 family member A1 | ALDH1A1 |
| 14. | P00734 | Coagulation factor II, thrombin | F2 |
| 15. | Q13822 | Ectonucleotidepyrophosphatase/phosphodiesterase  2 | ENPP2 |
| 16. | O14746 | Telomerase reverse transcriptase | TERT |
| 17. | P41597 | C-C motif chemokine receptor 2 | CCR2 |
| 18. | P35968 | Kinase insert domain receptor | KDR |
| 19. | P60568 | Interleukin 2 | IL2 |
| 20. | P11021 | Heat shock protein family A (Hsp70) member 5 | HSPA5 |
| 21. | P19320 | Vascular cell adhesion molecule 1 | VCAM1 |
| 22. | P14780 | Matrix metallopeptidase 9 | MMP9 |
