# Supplementary material for: Anti-hepatocellular carcinoma activity of Jacaranda mimosifolia through experimental validation and network pharmacology
Source: PLoS One. 2026 Apr 3;21(4):e0346325. doi: 10.1371/journal.pone.0346325 (PMC13048444; doi:10.1371/journal.pone.0346325)
Supplement: S1 File — (PDF) [file pone.0346325.s002.pdf]

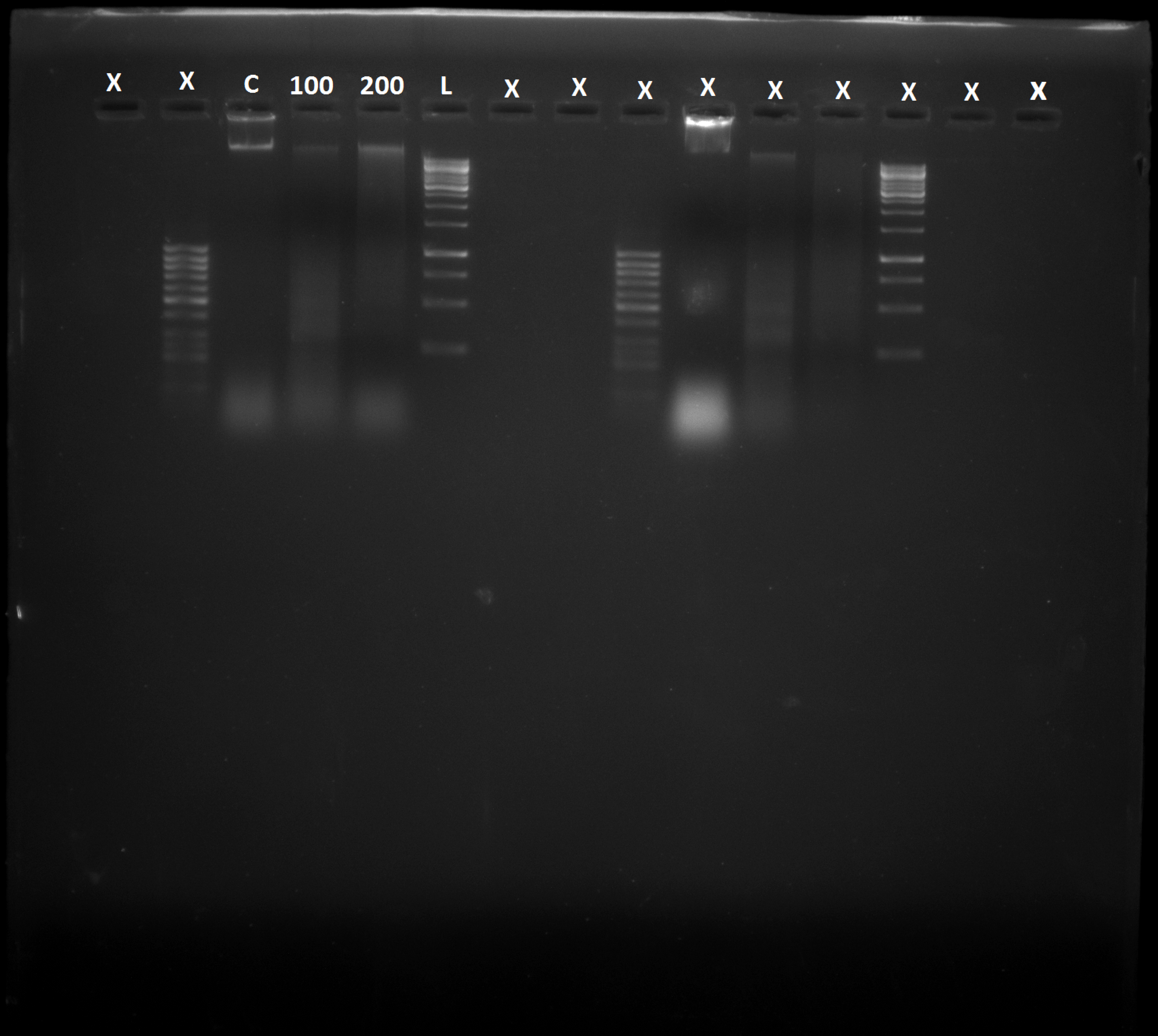

Figure 3 captured by UV transilluminator

C = Huh-7.5 cells as control
100 = Huh-7.5 cells treated with 100 µg/ml of *J. mimosifolia*
200 = Huh-7.5 cells treated with 200 µg/ml of *J. mimosifolia*
L = Ladder = MW marker = 100 bp
